# GPR108 Negatively Regulates TLR7 Signaling in Imiquimod‐Induced Psoriasiform Dermatitis

**DOI:** 10.1002/iid3.70346

**Published:** 2026-02-12

**Authors:** Wenwen Wang, Yuyan Zhang, Kainan Liao, Qiantong Xiang, Dandan Zang, Chunlin Cai, Fusheng Zhou, Haisheng Zhou

**Affiliations:** ^1^ Department of Biochemistry and Molecular Biology Anhui Medical University Hefei China; ^2^ Clinical Laboratory Mianyang Maternity and Child Healthcare Hospital Mianyang China; ^3^ Key Laboratory of Dermatology (Anhui Medical University), Ministry of Education Hefei China; ^4^ Department of Dermatology The Affiliated Hefei Hospital of Anhui Medical University Hefei China; ^5^ Center for Scientific Research Anhui Medical University Hefei China; ^6^ Department of Pathophysiology School of Basic Medical Sciences, Anhui Medical University Hefei China

**Keywords:** GPR108, inflammation, keratinocyte, macrophage, NF‐κB, psoriasis, TLR7

## Abstract

**Objective:**

Psoriasis, a common chronic inflammatory skin disease, is characterized by epidermal hyperplasia and inflammatory cell infiltration. While endosomal Toll‐like receptors (TLRs), particularly TLR7, are key drivers of psoriatic exacerbations, the regulatory mechanisms governing TLR7 activity in psoriasis remain incompletely understood. This study aims to investigate the role of G protein‐coupled receptor 108 (GPR108) in regulating TLR7 activity during imiquimod (IMQ)‐induced psoriasiform dermatitis.

**Methods:**

We established IMQ‐induced psoriasiform lesions in *Gpr108*‐null mice, as well as IMQ‐treated GPR108‐deficient keratinocyte and macrophage models. The psoriasis‐like phenotype was assessed in vivo using PASI scoring and H&E staining. Protein expression was examined by Western blotting, immunohistochemistry, and immunofluorescence. Additionally, RNA‐seq and flow cytometric analyses were performed to verify the involvement of the TLR7/NF‐κB signaling in regulating GPR108‐deficient macrophage polarization.

**Results:**

We found that *Gpr108* deficiency exacerbates IMQ‐induced psoriatic lesions in mice. Mechanistically, GPR108 deficiency enhances TLR7/MyD88/NF‐κB signaling in both keratinocytes and macrophages following imiquimod stimulation. This increased TLR7 activation promotes keratinocyte hyperproliferation and dysregulated differentiation, and alters the M1/M2 macrophage balance, leading to elevated production of cytokines, including TNF‐α and IL‐6.

**Conclusion:**

GPR108 functions as a negative regulator of TLR7 signaling in psoriasis.

## 1 Introduction

Psoriasis is a prevalent chronic inflammatory skin disease characterized by hyperplasia and aberrant differentiation of epidermal keratinocytes. The interaction between keratinocytes and infiltrating immune cells, such as macrophages, neutrophils, dendritic cells, and activated T cells, are thought to be important for psoriasis initiation and maintenance [1, 2, 3]. Toll‐like receptors (TLRs) have been implicated in these interactions and in the exacerbation of psoriasis [4, 5]. Inappropriate and excessive activation of the TLRs of the infiltrating macrophages of psoriatic lesions produces cytokines that foster the development of psoriatic lesions. Agonists of endosomal TLRs (TLR7, TLR8, and TLR9), promote M1 macrophage polarization [1, 6].

Keratinocytes play a pivotal role as the targets of pro‐inflammatory cytokines and are actively involved in the initiation of psoriasis [2]. Additionally, keratinocytes are known to express various TLRs. Activation of TLR signaling pathways, including both MyD88‐dependent and MyD88‐independent pathways, promotes the nuclear translocation of nuclear factor for kappa B (NF‐κB), which in turn induces the production of inflammatory cytokines [5]. Upregulation of TLR7 results in psoriatic exacerbation due to its activation of the TLR7/NF‐κB pathway in a MyD88‐dependent manner [7, 8]. This activation subsequently enhances the production of various pro‐inflammatory mediators including interleukin (IL)‐6, IL‐1, and tumor necrosis factor‐alpha (TNF‐α).

The synthetic TLR7 agonist, imiquimod (IMQ), is commonly employed to induce psoriasiform lesions in mouse skin. This is achieved by activating the TLR7/NF‐κB pathway in a MyD88‐dependent manner [7, 9]. Although the activation and functions of TLR7 are well understood, little attention has been paid to potential negative signaling pathways that might regulate the functions of TLR7 and maintain a balance between activation and repression of the TLR7 signaling pathway in psoriasis.

G protein‐coupled receptor 108 (GPR108), also referred as the lung seven‐transmembrane receptor 2 (LUSTR2), was initially identified as a member of the G protein‐coupled receptor superfamily through sequence analysis in 2007 [10]. GPR108 is mainly located on subcellular membranes, including the Golgi apparatus membrane and the membranes of vesicles. More recently, it has been recognized as an important receptor for adeno‐associated viruses (AAV) in relation to cellular immune responses [11]. We discovered that *Gpr108*‐null mice do not exhibit any conspicuous phenotypic characteristics [12]. Our study also demonstrated that GPR108 functions as a regulator of immune responses activated by TLRs through its antagonistic interaction with MyD88. Therefore, GPR108 potentially plays a crucial role in maintaining the balance between stimulatory and inhibitory TLR‐triggered immune responses, which suggests its involvement in inflammation‐related diseases by dysregulating NF‐κB activity. Particularly, understanding the pathological roles and mechanisms of GPR108 in psoriasis is of utmost importance.

In this study, we observed that GPR108 deficiency increases inflammation and the severity of the disease in IMQ‐induced psoriasiform skin lesions in mice. These findings prove that GPR108 plays crucial roles in the amplification and progression of psoriasis pathogenesis. Upon IMQ stimulation, GPR108 deficiency enhances the proliferation and differentiation of keratinocytes by activating the TLR7/NF‐κB signaling pathway in a manner dependent on MyD88 expression. Furthermore, the present study reveals that stronger inflammatory responses were observed in *Gpr108* ^−/−^ mice with IMQ‐induced psoriasiform lesions, which were accompanied by M1 polarization and the production of inflammatory cytokines. These findings indicate that GPR108 acts as a negative regulator of inflammation responses triggered by TLR7, emphasizing its potential as a therapeutic target for psoriasis treatment.

## 2 Materials and Methods

### 2.1 Mice

C57BL/6J mice were purchased from GemPharmatech Corporation (Nanjing, China). The *Gpr108*‐null mice (*Gpr108* ^−/−^mice), which were on a C57BL/6J background, were generously provided by the Center for Computational and Integrative Biology at Massachusetts General Hospital. The murine *Gpr108* locus containing 17 exons was deleted by using a BAC‐mediated homologous recombination method. The process of generating the *Gpr108*‐null mice has been previously described [12]. The primers P1/P2 and P3/P4 were used for performing PCR to detect mouse genotypes (Table S1). PCR analysis was performed initially to determine the genotype of mice. The results indicated that wild‐type mice, referred to as WT, had a 621 bp fragment, whereas knockout mice, named as KO, exhibited a 362 bp fragment (Figure S1A,B). All mice were bred and maintained under specific pathogen‐free conditions at the Animal Center of the School of Basic Medical Sciences of AHMU. All animal experiments were conducted following protocols approved by the Institutional Animal Care and Use Committee of AHMU (Approval No.: LLSC20200007).

### 2.2 IMQ‐Induced Psoriasis

Seven‐ to ten‐week‐old male mice (WT, *n* = 6; KO, *n* = 6) received a daily dose of 60.0 mg IMQ cream (5%, Med‐shine Pharma, Chengdu, China) on a 2 cm × 2 cm patch of shaved dorsal skin for 5 consecutive days. Control groups consisted of WT and KO mice, with *n* = 6 per group. All mice were euthanized on Day 5, Psoriasis Area and Severity Index (PASI) scores were analyzed, and samples were collected. For downstream experiments, skin lesional tissues from three mice per group were sectioned, and proteins were extracted from the lesional tissues of three additional mice per group. The PASI scores, which consisted of measurements or inflammation, including skin erythema, scaling, and thickness, was usually used to monitor and grade the severity of the psoriasis‐like lesions on Day 3 and 5. Each parameter was scored independently on a five‐point scale from 0 to 4, namely: 0, none; 1, slight; 2, moderate; 3, marked; and 4, very marked [13].

### 2.3 Cells and Cell Culture

Primary bone marrow‐derived macrophages (BMDMs) were prepared as described [12]. Cells were cultured and differentiated in the presence of 20 ng/mL of macrophage colony‐stimulating factor (M‐CSF, Novoprotein, CB34) in Dulbecco's Modified Eagle Medium (DMEM) with 10% fetal bovine serum (FBS; ExCell, FSP500) and penicillin‐streptomycin (PS; Beyotime, Shanghai, China) at a concentration of 100 U/mL and 100 μg/mL, respectively. BMDMs were treated with 10 μg/mL of IMQ (InvivoGen) for 24 or 48 h.

The human immortalized keratinocytes (HaCaT), the human monocyte line (THP1), and the human embryonic kidney cell line (HEK‐293T) were purchased from the Cell Resource Center of the Institute of Basic Medical Sciences at Chinese Academy of Medical Sciences & Peking Union Medical College. HaCaT and HEK‐293T were cultured in DMEM supplemented with 10% FBS and PS. To induce cell differentiation, HaCaT cells were treated with or without IMQ (2.5 µg/mL) in D‐KSFM medium (Gibco 10744019) for 3 days. THP‐1 cells were cultured in RPMI‐1640 medium supplemented with 10% FBS and PS. To induce differentiation into M0 macrophages, the THP‐1 cells were treated with 100 ng/ml of phorbol 12‐myristate 13‐acetate (PMA) for 48 h. GPR108 knockout cell clones, including *GPR108* ^−/−^ HaCaT and *GPR108* ^−/−^ THP‐1, were generated by using the CRISPR/Cas9 technique. To inhibit the MyD88 pathway, cells were subjected to treatment with the MyD88 inhibitor (TJ‐M2010‐5, TJ‐M, 25 µM) for a period of 3 h prior to activation. All cells were cultured at 37°C under an atmosphere of 5% CO_2_.

### 2.4 RNA Isolation, Reverse‐Transcribed Quantitative PCR (RT‐qPCR) and RNA Sequencing Analysis (RNA‐Seq)

THP‐1 cells were treated with or without IMQ at a concentration of 10 µg/mL for different times. Total RNA was extracted from IMQ‐treated *GPR108* ^+/+^ and *GPR108* ^−/−^ THP‐1 cells using Trizol (Invitrogen, 15596018). RNA was reverse‐transcribed to cDNA using the RevertAid First Strand cDNA Synthesis Kit (Thermo Scientific, K1622). Quantitative PCR was performed using SYBR qPCR Master Mix (TOLOBIO, 22204) with a LightCycler 480 II Real Time Fluorescence Quantification System (Roche, LC480Ⅱ). Gene expression was calculated using the $2^{‐∆∆C_{t}}$ method relative to the GAPDH. All primers listed in Table S1 were synthesized by Tsingke Biotechnology Corporation (Beijing, China).

Total RNA extraction and purification, library construction, and sequencing were carried out by LC‐Bio Technology (Hangzhou, China).

### 2.5 Cell Viability Assay

Cell viability was detected by using the Cell Counting Kit‐8 (CCK‐8, APExBIO, K1018) in accordance with the manufacturer's instructions. The absorbance at 450 nm was measured with a microplate reader (Bio‐Rad), using wells without cells as blanks and untreated cells as negative controls.

### 2.6 Western Blot

Cell and skin lysate proteins were resolved by SDS‐PAGE, transferred to polyvinylidene difluoride membranes, and immunoblotted [14]. The antibodies used in this study included TLR7 (Proteintech, 17232‐1‐AP), MyD88 (ZENBIO, 340629), phospho‐P65 (p‐P65, Santa Cruz, sc‐136548), P65 (Cell Signaling Technology, CST, 8242S), Involucrin (IVL, Santa Cruz, sc21748), IVL (Proteintech, 83649‐5‐RR), Keratin 1 (K1, Abcam, ab93652), Keratin 5 (K5, Abcam, ab53121), GAPDH (DUONENG‐BIO, AB‐010301), horseradish peroxidase (HRP)‐conjugated goat anti‐mouse IgG (CST, 7076S), and HRP‐conjugated goat anti‐rabbit IgG (CST, 7074S). Signals were detected with an enhanced chemiluminescence (ECL) solution (Advansta, K‐12045‐D50) and the images were visualized using a GEL imaging system (GE, AI600 RGB).

### 2.7 Histopathological Analysis, Immunohistochemistry and Immunofluorescent Staining

Hematoxylin‐eosin (H&E) staining, immunohistochemistry, and immunofluorescence staining were performed using standard techniques as previously described [14]. Epidermal thickness was calculated by averaging over three randomly selected areas from each mouse. The antibodies used for immunohistochemistry included those specific for TLR7 (Proteintech, 17232‐1‐AP), MyD88 (ZENBIO, 340629), and p‐P65 (Santa Cruz, sc‐136548).

The following primary antibodies were used for immunofluorescence staining: rabbit anti‐mouse F4/80 (CST, 32325S), mouse anti‐mouse CD206 (Santa Cruz, sc‐376108), mouse anti‐mouse CD86 (Santa Cruz, sc‐28347), rabbit anti‐mouse K1 (Abcam, ab93652), rabbit anti‐mouse K5 (Abcam, ab53121), and mouse anti‐mouse IVL (Santa Cruz, sc21748). The secondary antibodies included Alexa Fluor 647‐conjugated goat anti‐mouse IgG (H + L) (CST, 4410S), Alexa Fluor 488‐conjugated goat anti‐mouse IgG (H + L) (CST, 4408S) and Alexa Fluor 594‐conjμgated goat anti‐rabbit IgG (H + L)(CST, 8889S). Nuclei were stained with Hoechst 33342 (Biosharp, BS117‐25 mg).

### 2.8 Flow Cytometric Analysis

Multi‐color flow cytometry analysis was performed to identify BMDMs through the assessment of F4/80^+^ and CD11b^+^ cell marker expression. The antibodies used for labeling were fluorochrome‐conjugated, namely PE‐F4/80 (BD, 565410) and FITC‐CD11b (Invitrogen, 11‐0112‐82). To examine the polarization of BMDMs, dead cells were initially stained with FVS450 (Fixable Viability Stain 450, BD, 562247). Subsequently, a fluorochrome‐conjugated monoclonal antibody (BB700‐CD86, BD, 742120) was used to label the surface markers of M1 macrophages. Fixed and permeabilized cells were stained with APC‐CD206 (Invitrogen, 17‐2061‐82) to identify markers associated with M2 macrophages.

### 2.9 Statistics

Data are presented as mean ± standard deviation (SD) and were analyzed using GraphPad Prism 9.0.0 software. Two‐sided unpaired Student's *t*‐test or Two‐way ANOVA with Tukey's post test was used to compare the two groups; One‐way ANOVA with Tukey's post‐test or Kruskal‐Wallis test was used to perform comparisons among multiple groups.

## 3 Results

### 3.1 *Gpr108* Deletion Exacerbates IMQ‐Induced Psoriasiform Lesions in Mice

**Figure 1:**
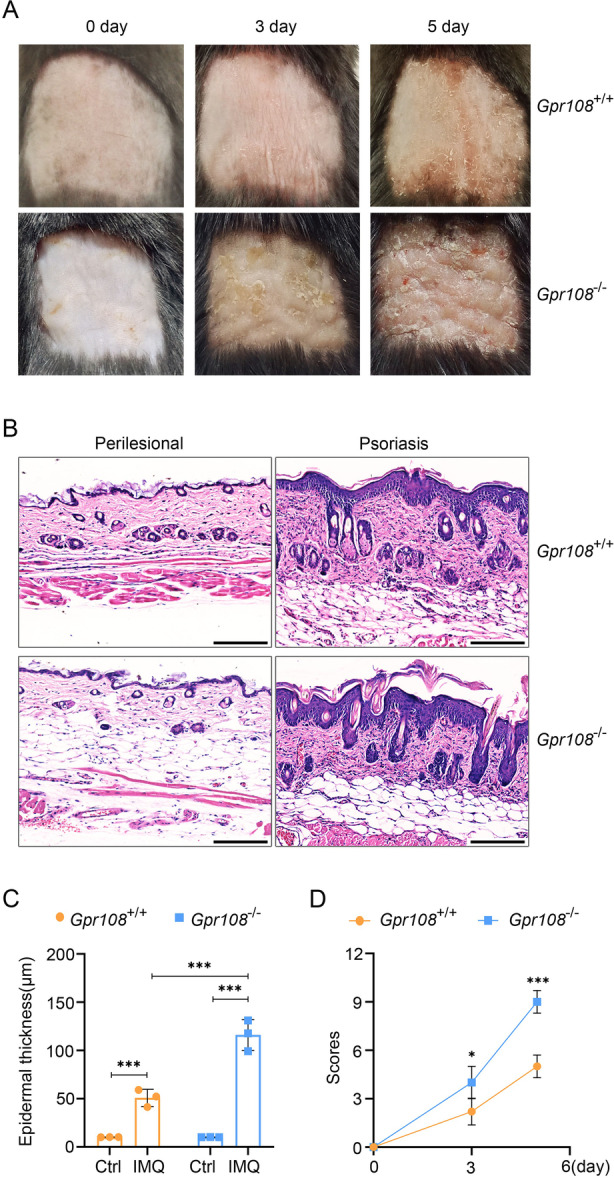
*Gpr108* deletion exacerbates IMQ‐induced psoriasiform skin lesions in mice. (A) Representative examples of dorsal skin from *Gpr108* ^+/+^ (WT) and *Gpr108* ^−/−^ (KO) mice treated with IMQ for 3 and 5 days. (B) Representative images of dorsal skin sections stained by H&E. Scale bars = 200 µm. (C) Statistical analysis of measurements of epidermal thickness. Data are shown as mean ± SD (*n* = 3/group). ***p < 0.001. (D) The total PASI scores of the skin tissues. The PASI scores for the IMQ‐induced psoriasiform skin lesions in mice were scored on Days 3 and 5. Data are shown as mean ± SD. *p < 0.05; **p < 0.01. For panel (C), a two‐way ANOVA with Tukey's post hoc test was used. For panel (D), a two‐sided unpaired Student's *t*‐test was performed. ANOVA, analysis of variance; H&E, hematoxylin‐eosin; IMQ, imiquimod; PASI, Psoriasis Area and Severity Index.

To further investigate the potential role of GPR108 in psoriasis, psoriasiform lesions were created in *Gpr108*‐null mice using IMQ induction. During the second and third days of IMQ application, an erythematous rash, scales, and thickening of the skin were observed on the dorsal area of both C57BL/6J mice (WT) and *Gpr108*‐null mice (KO). The severity of these manifestations increased from days 2‐3 until the completion of the experiment. Typical examples were shown in Figure 1A. The psoriasiform lesions, accompanied by inflammation, were noticeably more visible on the backs of KO mice and increased scaling and pronounced swelling was observed. The analysis of the H&E‐stained sections from the IMQ‐treated skin showed epidermal parakeratosis and increased epidermal thickening in the dorsal skin (Figure 1B). H&E staining demonstrated a significant increase in epidermal thickness and infiltration of cells in the dermis of KO mice compared to WT mice (Figure 1B,C). The PASI scores, assessed on Days 3 and 5, illustrated significantly higher scores for the KO mice compared to the WT mice (Figure 1D). These findings suggest that GPR108 deficiency promotes the development of psoriasiform lesions induced by IMQ.

### 3.2 GPR108 Deficiency Results in Increased Proliferation and Alters Differentiation of Keratinocytes Induced by IMQ

**Figure 2:**
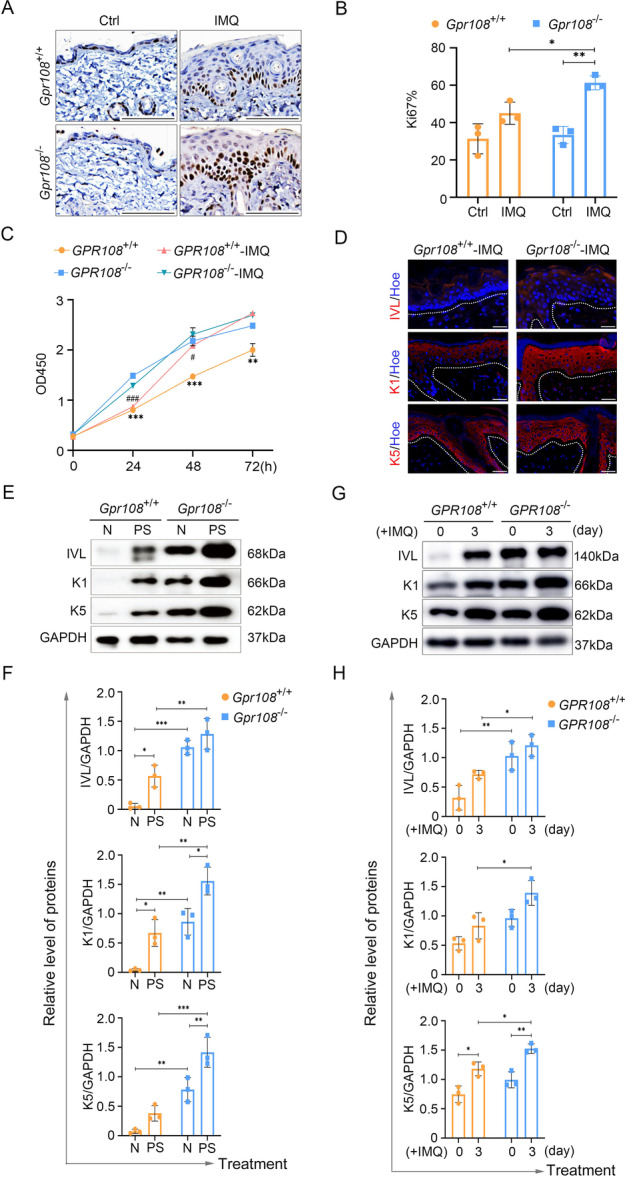
GPR108 deficiency contributes to proliferation and differentiation of keratinocytes in the presence of IMQ. (A) Immunohistochemistry labeling of Ki67 in mouse skin samples (*n* = 3/group). Scale bars = 100 μm. (B) Statistical analysis of Ki67^+^ cells per field. Randomize three images and calculate the ratio of the number of positive cells to the total number of cells in the same area. (C) Proliferative capacity assay of HaCaT treated with IMQ (1 µg/mL) for 24, 48, and 72 h. ***p < 0.001, **p < 0.01, *GPR108* ^+/+^ versus *GPR108* ^−/−^ HaCaT cells; ^###^ *p* < 0.001, ^#^ *p* < 0.05, IMQ‐*GPR108* ^+/+^ versus IMQ‐*GPR108* ^−/−^ HaCaT cells. (D) Immunofluorescence analysis of differentiated markers in skin tissues of mice. Scale bars = 20 µm. (E, F) Immunoblotting analysis for expression of the differentiated markers in skin tissues. N, normal skin tissues of mice; PS, IMQ‐induced psoriasiform lesions of mice. (G, H) Immunoblotting analysis for expression of the differentiated markers in HaCaT treated with or without IMQ (2.5 µg/mL) for 3 days. Data are shown as mean ± SD. *p < 0.05; **p < 0.01. The experiments were repeated three times. For panels (B, F, H), a two‐way ANOVA with Tukey's posttest was performed. For panel (C), a two‐sided unpaired Student's *t* test was used. ANOVA, analysis of variance; GAPDH, glyceraldehyde 3‐phosphate dehydrogenase; IMQ, imiquimod.

The increased epidermal thickness was mainly attributable to excessive proliferation of keratinocytes, which is a defining characteristic of psoriasis. As shown in Figure 2A, an increased number of keratinocytes showed Ki67 positive labeling in the epidermis of IMQ‐induced psoriasiform lesions. The *Gpr108* ^−/−^ mice had a significantly higher Ki67‐labeling index compared to *Gpr108* ^+/+^ mice (Figure 2B). To investigate the role of GPR108 in keratinocyte proliferation in vitro, we used CRISPR/Cas9 to disrupt the GPR108 gene locus in HaCaT cells, as detailed in the Supporting Data (Figure S1C). The proliferation capacity of *GPR108* ^−/−^ HaCaT cells was found to be significantly higher than *GPR108* ^+/+^ HaCaT cells in CCK‐8 assays. Importantly, this enhanced proliferation was sustained even with the addition of IMQ (Figure 2C).

Aberrant differentiation of epidermal keratinocytes is a prominent feature of psoriasis. Various specific molecular markers that indicate different layers of the epidermis are commonly used to identify keratinocyte differentiation. For instance, keratin 5 or 10 (K5 or K10) is predominantly expressed in the basal layer of the epidermis, whereas keratin 1 (K1) is primarily expressed in the spinous and granular layers. In addition, IVL is expressed in the stratum corneum. Both the histological immunofluorescence analysis and immunoblotting analysis showed that the expression of K1, K5, and IVL significantly increased in IMQ‐induced psoriasiform skin lesions of the KO mice compared to those of the WT mice (Figure 2D–F). Furthermore, in the skin tissue of the WT mice, the expression of these proteins was almost undetectable, whereas in the skin tissue of the KO mice, these proteins showed relatively high expression levels. Additionally, the expressions of K1, K5 and IVL in *GPR108* ^−/−^ HaCaT cells were significantly higher than those in *GPR108* ^+/+^ HaCaT cells, which were treated by IMQ (Figure 2G,H).

### 3.3 GPR108 Deficiency Contributes to the Activation of the TLR7/MyD88/P65 Signaling Pathway in Psoriatic Keratinocytes

**Figure 3:**
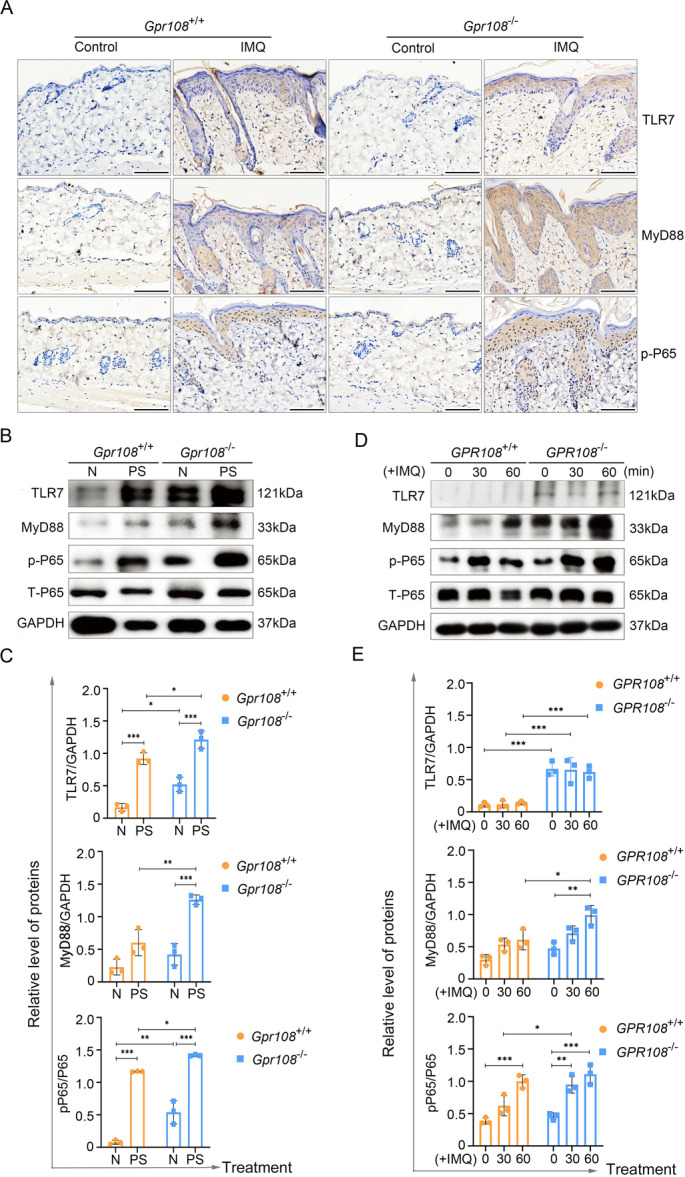
TLR7, MyD88, and p‐P65 are upregulated in psoriatic lesions in IMQ‐induced psoriasiform lesions in mice and IMQ‐treated keratinocytes. (A) Immunohistochemistry analysis for the expression of the TLR7 and MyD88, along with the levels of the p‐P65 in mouse skin biopsies. Scale bars = 100 µm. (B, C) Immunoblotting analysis of levels of TLR7, MyD88, and p‐P65 in the skin tissues from the IMQ‐induced psoriasiform lesions in mice. (D, E) Western blot analysis of TLR7, MyD88, p‐P65, and total P65 (T‐P65) in HaCaT cells treated with IMQ (10 µg/mL) for 30 and 60 min. The experiments were repeated three times. Significance was determined using two‐way ANOVA with Tukey's posttest. ANOVA, analysis of variance; GAPDH, glyceraldehyde 3‐phosphate dehydrogenase; IMQ, imiquimod.

To investigate the potential involvement of TLR7 signaling in psoriasis, an immunohistochemical analysis was performed to confirm the presence of TLR7, MyD88, and p‐P65 in psoriatic lesions, perilesional tissues, and healthy skin. As shown in Figure 3A, the psoriasiform lesions of KO mice exhibited higher presence of TLR7, MyD88, and p‐P65 compared to the WT mice. Proteins were extracted from the IMQ‐induced psoriasiform lesions and subjected to Western blotting analysis. The results were found to be consistent with the findings from the immunohistochemical analyses (Figure 3B,C).

The presence of TLR7, MyD88, and p‐P65 was further detected in both *GPR108* ^+/+^ and *GPR108* ^−/−^ HaCaT cells after treatment with IMQ for 30 and 60 min. The results showed a significant increase in the expression of MyD88 in both *GPR108* ^+/+^ and *GPR108* ^−/−^ HaCaT cells treated with IMQ, accompanied by an increase of p‐P65 (Figures 3D,E). Additionally, *GPR108* ^−/−^ HaCaT cells exhibit increased sensitivity to IMQ compared to *GPR108* ^+/+^ HaCaT cells, due to the time‐dependent upregulation of MyD88, and p‐P65.

### 3.4 Deficiency of GPR108 Leads to Abnormal Cytokine Production Associated With the Activation of the TLR7/MyD88/P65 Signaling Pathway in Macrophages

**Figure 4:**
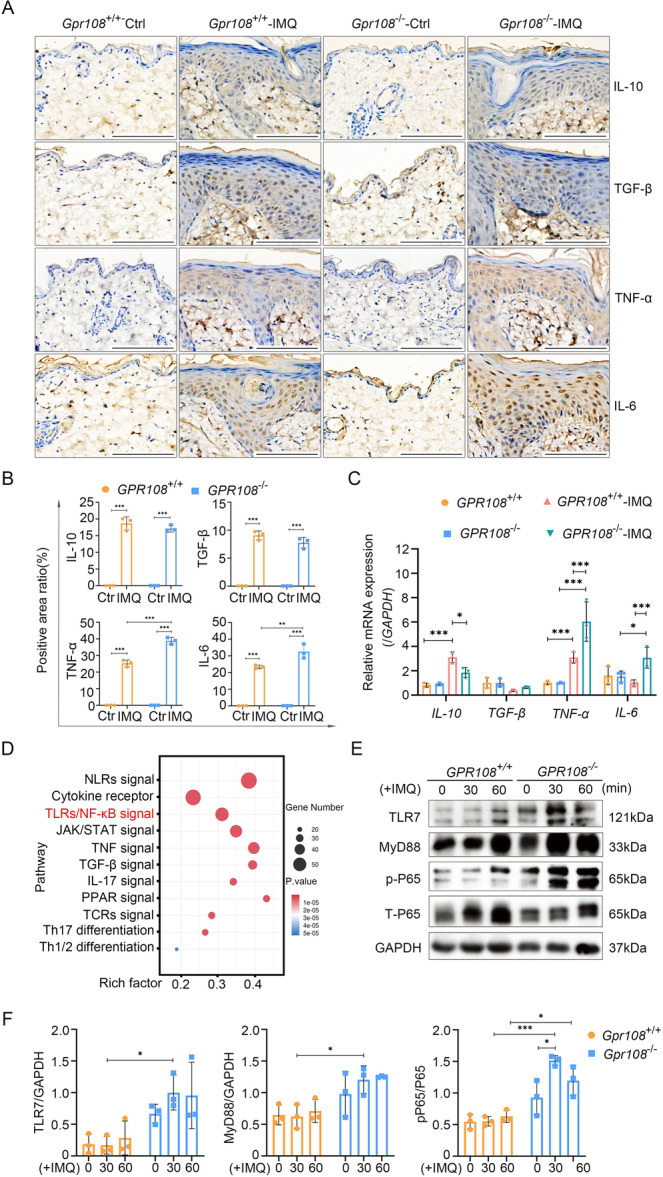
GPR108 deficiency leads to an aberrant cytokine production associated with the activation of the TLR7/MyD88/p‐P65 pathway in macrophages. (A) Immunohistochemistry analysis of IL‐10, TGF‐β, TNF‐ɑ, and IL‐6 in skin biopsies of mice, including the normal skin tissues and the IMQ‐induced psoriasiform lesions of mice. Scale bars = 100 µm. (B) Quantitative analysis of immunohistochemical optical density from panel (A). Data are shown as mean ± SD (3 fields/group). **p < 0.01; ***p < 0.001. Significance was determined using two‐way ANOVA with Tukey's posttest. (C) RT‐qPCR analysis of IL‐10, TGF‐β, TNF‐ɑ, and IL‐6 in THP‐1 cells treated with IMQ (10 µg/mL) for 48 h. (*n* = 3/group). (D) Signal pathway analysis for RNA‐seq data from THP‐1 cells treated with IMQ. (E, F) Western blotting analysis of TLR7, MyD88, and p‐P65 in THP‐1 cells treated with IMQ (10 µg/mL) for 30 and 60 min. Data are shown as mean ± SD. *p < 0.05; **p < 0.01; ***p < 0.001. The experiments were repeated three times. Significance was determined using two‐way ANOVA with Tukey's posttest. ANOVA, analysis of variance; GAPDH, glyceraldehyde 3‐phosphate dehydrogenase; IL, interleukin; IMQ, imiquimod; RT‐qPCR, reverse‐transcribed quantitative polymerase chain reaction; TGF‐β, transforming growth factor‐β; TNF‐ɑ, tumor necrosis factor‐ɑ.

To understand why the deletion of *Gpr108* elicits more pronounced inflammatory responses in IMQ‐induced psoriasiform skin lesions, an exploration of the peripheral blood lymphocytes was undertaken. T cells are key participants in psoriatic inflammation, that release pro‐inflammatory cytokines and contribute to the exaggerated immune response and inflammation in psoriasis lesions. T cells from the peripheral blood of both WT and KO mice were analyzed by flow cytometry, but no significant differences in the proportions of CD3^+^, CD3^+^CD4^+^, and CD3^+^CD8^+^ T cells between WT and KO mice were found (Figure S2). Because myeloid cells can play a significant role in the development and maintenance of psoriatic lesions, *GPR108* was inactivated in human THP‐1 cells using the CRISPR/Cas9 methods as described in the supporting data (Figure S1D). From the RNA‐seq data, we identified a total of 195 inflammation‐related genes that exhibited differential expression in GPR108‐deficient cells compared to WT cells. Among these, 55 genes displayed upregulated expression, while 23 genes showed downregulated expression (Figure S3). IHC analysis was performed to detect expression of IL‐6, TNF‐ɑ, IL‐10, and TGF‐β in the IMQ‐induced psoriasiform lesions. The expression levels of IL‐10 and TGF‐β showed no significant differences (Figure 4A,B). Interestingly, both IL‐6 and TNF‐ɑ levels were significantly elevated in the psoriasiform lesions induced by IMQ, as compared to normal skin. The deletion of GPR108 resulted in higher levels of IL‐6 and TNF‐ɑ in the lesions. RT‐PCR analysis was performed to further investigate the expression levels of these cytokines in *GPR108* ^−/−^ THP‐1 cells in comparison to *GPR108* ^+/+^ cells. We observed IMQ treatment significantly increased IL‐6 and TNF‐ɑ expression in *GPR108* ^−/−^ THP‐1 cells compared to *GPR108* ^+/+^ THP‐1 cells. However, no notable difference was observed in the expression of transforming growth factor‐β (TGF‐β) between the two types of cells treated with or without IMQ. Interestingly, we found that GPR108 deficiency significantly reduced IL‐10 expression in THP‐1 cells (Figure 4C).

To determine whether TLR7 and its downstream NF‐κB signaling pathway regulates cytokine production of *GPR108* ^−/−^ macrophages induced by IMQ, we conducted RNA‐seq analysis using *GPR108* ^−/−^ THP‐1 cells. The results showed that there were 50 genes associated with TLR7 and its downstream NF‐κB signaling pathway (Figure 4D). Western blotting analysis identified a significant increase in the expression of TLR7 and MyD88 in THP‐1 cells treated with IMQ, accompanied by an upregulation in p‐P65 levels (Figure 4E,F). More important, GPR108 deficiency is beneficial for the activation of the TLR7/MyD88/NF‐κB signals, even in the absence of IMQ treatment.

### 3.5 Altered M1/M2 Macrophage Ratios Induced by IMQ in the Context of GPR108 Deficiency Cause Abnormal Cytokine Production

**Figure 5:**
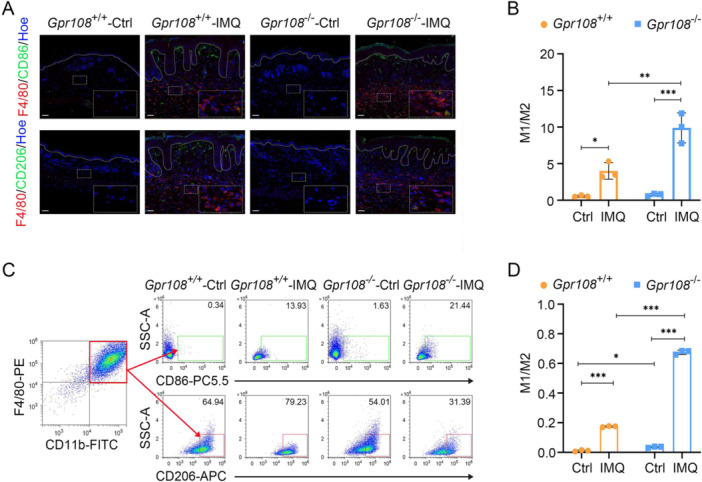
GPR108 deficiency is more favorable for the polarization of M1 macrophages induced by IMQ. (A) Immunofluorescence analysis of M1 and M2 macrophages in skin biopsies of mice. M1, F4/80^+^ (red) CD86^+^ (green); M2, F4/80^+^ (red) CD206^+^ (green); Nuclear staining was performed using Hoechst 33342 (Hoe). Scale bars = 50 µm. (B) The ratio of M1 and M2 macrophages in skin biopsies of mice. Ctrl, normal skin tissues; IMQ, IMQ‐induced psoriasiform lesions. (C) Representative flow cytometry plots of BMDMs polarization. The populations of M1 (F4/80^+^CD11b^+^CD86^+^) and M2 (F4/80^+^CD11b^+^CD206^+^) macrophages derived from BMDMs treated with IMQ (10 µg/mL) for a duration of 48 h. (D) Statistical analysis of the ratio between M1 and M2 derived from BMDMs through IMQ induction. Data are shown as mean ± SD (*n* = 3/group). *p < 0.05, **p < 0.01, ***p < 0.001. Significance was measured using two‐way ANOVA with Tukey's posttest. ANOVA, analysis of variance; BMDM, bone marrow‐derived macrophages.

Because IL‐6 and TNF‐α expression is primarily associated with M1 macrophages, we performed immunofluorescence to analyze both M1 and M2 macrophage populations in IMQ‐induced psoriasiform skin lesions. Figure 5A illustrates the presence of CD86 + F4/80+ cells, identified as M1 macrophages, and CD206 + F4/80+ cells, identified as M2 macrophages, in the dermal layers of skin tissues. Analysis of WT mice revealed M1/M2 macrophage ratios of 0.56 ± 0.13 in normal skin and 4.01 ± 2.03 in psoriasiform lesions. In contrast, KO mice exhibited M1/M2 macrophage ratios of 0.76 ± 0.23 in normal skin and 9.89 ± 3.49 in psoriasiform lesions (Figure 5B). Notably, both WT and KO mice exhibited significantly higher M1/M2 ratios in psoriasiform lesions compared to normal skin (*p* = 0.0292 and *p* < 0.001, respectively). Furthermore, WT mice exhibited lower M1/M2 ratios in psoriasiform lesions compared to KO mice (*p* = 0.0013).

A flow analysis was performed to assess the differentiation of primary bone marrow‐derived macrophages (BMDMs) after IMQ treatment. As shown in Figure 5C, the percentage of M1 (F4/80^+^CD11b^+^CD86^+^CD206^−^) macrophages in WT mice was observed to be 0.69% ± 0.42%, whereas M2 (F4/80^+^CD11b^+^CD86^−^CD206^+^) macrophages constituted 63.33% ± 13.12%. In contrast, KO mice displayed a higher percentage of M1 macrophages (1.77% ± 0.12%), while the average percentage of M2 macrophages was 50.22% ± 3.29%. After induction by IMQ, *Gpr108* ^+/+^ BMDMs consisted of 13.13% ± 0.92% M1 macrophages and 75.41% ± 6.12% M2 macrophages. In contrast, *Gpr108* ^−/−^ BMDMs contained 20.97% ± 0.41% M1 macrophages and 31.04% ± 0.59% M2 macrophages. Compared to the control groups, both *Gpr108* ^+/+^ and *Gpr108* ^−/−^ BMDMs demonstrated significantly increased ratios of M1/M2 when exposed to IMQ treatment (*p* < 0.001) (Figure 5D). IMQ‐induced *Gpr108* ^−/−^ BMDMs exhibited a higher ratio of M1/M2 compared to the IMQ‐induced *Gpr108* ^+/+^ BMDMs (*p* < 0.001).

### 3.6 GPR108 Deficiency Contributes to the Activation of TLR7 Signaling in Keratinocytes and Macrophages via the MyD88‐Dependent Pathway

**Figure 6:**
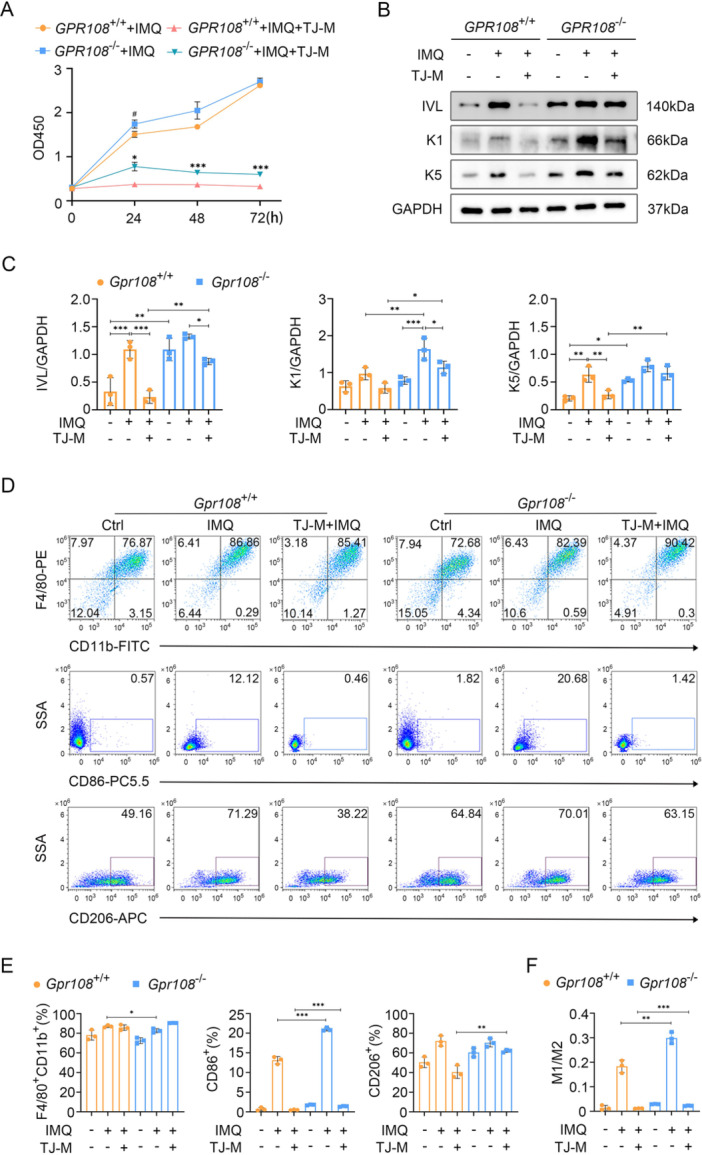
GPR108 deficiency enhances the activation of TLR7 signaling in keratinocytes and macrophages through the MyD88‐dependent pathway. (A) Proliferative capacity assay for HaCaT cells exposed to the MyD88 inhibitor. After pretreating HaCaT cells with TJ‐M2010‐5 (TJ‐M, 25 µM) for 3 h, both *GPR108* ^+/+^ and *GPR108* ^−/−^ HaCaT cells were subjected to 1 µg/mL IMQ treatment for 24, 48, and 72 h. ^#^ *p* < 0.05, (*GPR108* ^−/−^ + IMQ) versus (*GPR108* ^+/+^ + IMQ); ***p < 0.001 (*GPR108* ^−/−^ + IMQ + TJ‐M) versus (*GPR108* ^+/+^ + IMQ + TJ‐M). (B, C) Immunoblotting analysis for expression of the differentiated markers in HaCaT. The MyD88 inhibitor modulates the effect of IMQ‐induced cell differentiation. HaCat cells, which were pretreated with or without TJ‐M (25 µM) for a duration of 3 h, were cultured in a medium containing 2.5 µg/mL IMQ for 3 days. (D–F) Representative flow cytometry plots and statistical analysis of BMDMs polarization. BMDMs were cultured in a medium lacking M‐CSF and subjected to a 3‐h pretreatment with or without TJ‐M (25 µM). Subsequently, the cells were stimulated with IMQ (10 µg/mL) for a 48‐h duration (*n* = 3/group). Data are shown as mean ± SD. *p < 0.05, **p < 0.01, ***p < 0.001. The experiments in were repeated three times. For panel (C), a two‐way ANOVA with Tukey's posttest was used. For panels (A, E, and F), a two‐sided unpaired Student's *t* test was performed. ANOVA, analysis of variance; BMDM, bone marrow‐derived macrophages; IMQ, imiquimod.

To confirm that the activation of the TLR7 signaling pathway depends on the MyD88 pathway, we attempted to use a MyD88 inhibitor (TJ‐M2010‐5) to block the TLR7 signaling pathway and observe the resulting effects on the proliferation and differentiation of keratinocytes, as well as the influence on macrophage polarization. As shown in Figure 6A, cell proliferation was enhanced under IMQ treatment, but the MyD88 inhibitor significantly inhibited cell proliferation, and this inhibition was more significant in GPR108^+/+^ HaCaT cells. Using the MyD88 inhibitor in HaCaT cells, we observed the suppression of IMQ‐induced effects on cell differentiation (Figure 6B,C). The findings indicate that IMQ is prone to activating the TLR7 signal in GPR108‐deficient keratinocytes through the MyD88‐dependent pathway.

Meanwhile, pre‐treatment of BMDMs with the MyD88 inhibitor did not affect the population of F4/80^+^CD11b^+^ cells induced by IMQ (Figure 6D,E). The population of F4/80^+^CD11b^+^CD86^+^ cells (M1 macrophages) significantly decreased after a 3‐h treatment of TJ‐M2010‐5 in both *Gpr108* ^−/−^ and *Gpr108* ^+/+^ BMDMs. The M1/M2 ratio of macrophages derived from BMDMs treated with IMQ exhibited a significant decline in responsiveness subsequent to pre‐treatment with the MyD88 inhibitor (Figure 6F). These findings are consistent with the view that activation of the IMQ‐induced TLR7 signal in macrophages polarization is dependent on the MyD88 pathway.

## 4 Discussion

GPR108, initially identified and examined in human lung tissues [10], has seven transmembrane helices and participates in a multitude of vital cellular signaling pathways. Recent studies have reported that GRP108 functions as a conserved AAV entry factor, contributing to cellular immune responses to AAV [11, 15]. GRP108 also plays a significant role in regulating NF‐κB signaling [12, 16], suggesting its potential involvement in inflammation‐related diseases by dysregulating NF‐κB activity. We have demonstrated that GPR108 acts as a negative regulator of immune responses triggered by TLRs through its antagonistic interaction with MyD88 [11]. However, less is known about the functions of GPR108 in inflammation‐related diseases. Psoriasis is a chronic inflammatory disease associated with the activation of TLR signaling pathways [5, 17, 18, 19]. IMQ, an agonist of TLR7, is widely used to induce psoriasiform lesions in mice. As such, these IMQ‐induced psoriasiform lesions recapitulate features of the human psoriasis, thereby supporting the notion that they serve as an effective model for examining the functions of GPR108 and its interaction with TLR7 in the context of psoriasis.

Interestingly, the psoriatic lesions induced by IMQ in *Gpr108*‐null mice exhibited more significant skin manifestations compared to those observed in WT mice. Gross morphological features included increased scaling, pronounced swelling, a significant increase in epidermal thickness, as well as greater total PASI scores. A higher proportion of proliferating cells, as judged by the proportion of nuclei expressing Ki67 antigen, was observed, consistent with perspectives that implicate keratinocyte dysregulation both in the onset of psoriasis as well as the propagation under the influence of a multimolecular network coordinated by cytokines [2, 14, 20]. It is well known that the key features of psoriasis are hyper‐proliferation and aberrant differentiation of keratinocytes, resulting in scaling and increased thickness of the epidermis [21, 22]. In the present study, CCK‐8 analysis identified a role for GPR108 in keratinocyte proliferation. Deficiency of GPR108 stimulated proliferation of keratinocytes induced by IMQ treatment compared to WT keratinocytes. In addition, immunofluorescence analysis of IMQ‐induced psoriasiform lesions and immunoblotting analysis of cells demonstrated that the deficiency of GPR108 more readily led to abnormal differentiation of keratinocytes induced by IMQ treatment, in contrast to WT keratinocytes. Therefore, GPR108 deficiency is beneficial for the hyperproliferation of keratinocytes induced by IMQ treatment. Also, the IMQ treatment results in a disturbed differentiation of keratinocytes (parakeratosis), all of which match the characteristics of the histological structure of plaque‐type psoriasis.

In psoriasis, inflammation leads to increased blood flow and the characteristic erythema and swelling observed in plaques, and significantly contributes to the development and progression of psoriasis. Although T lymphocytes have been implicated in the complex feedback mechanisms of psoriasis pathophysiology [23], no differences were observed in peripheral blood T cells between KO mice and WT mice in this study. Instead, a substantial dysregulation in the myeloid compartment was observed with increased representation of M1 macrophages. Given the crucial roles of macrophages in the development and maintenance of psoriatic lesions, the release of additional cytokines by macrophages is expected to recruit more monocytes to the site of inflammation and perpetuate the cycle of inflammation and keratinocyte proliferation [1, 24]. The present study reveals that GPR108 deficiency results in more prominent inflammatory reactions in IMQ‐induced psoriasiform skin lesions, associated with elevated levels of IL‐6 and TNF‐ɑ in the lesions, as compared to the levels observed in WT mice. TNF‐ɑ and IL‐6 are considered the primary pro‐inflammatory cytokines produced by M1 macrophages and play a crucial role in the development of psoriasis [25, 26]. TLR7/TLR8 activation promotes M1 macrophage or inflammatory macrophage polarization, which further amplifies the skin inflammation [17, 18, 24]. KO mice in this study exhibited higher M1/M2 ratios in psoriasiform lesions compared to WT mice, and IMQ‐induced *Gpr108* ^−/−^ BMDMs exhibited a higher M1/M2 ratio compared to the IMQ‐induced *Gpr108* ^+/+^ BMDMs. M2 macrophages can be divided into subtypes (M2a‐2d) under various stimulation conditions [27], but we were unable to detect any differences in subtypes of M2 macrophages. GPR108 deficiency appears to exacerbate the inflammatory response in psoriatic lesions by exacerbating the pro‐inflammatory properties of macrophages.

Activation of the signal pathway mediated by TLR7 is required for the development of psoriasiform lesions induced by IMQ [7, 27, 28, 29]. TLR7, as an endosomal receptor, transmits signals through the MyD88 pathway and its associated downstream molecules to induce various immune responses. In macrophages, NF‐κB acts downstream of MyD88 in the TLR7 signaling cascade [30, 31]. Lu et al. reported that IMQ induces the expression of M1 macrophage cytokines in psoriasiform lesions of mice and also increases the M1/M2 ratio [18]. Both exogenous and endogenous ligands of TLR7, TLR8, and TLR9 can activate the polarization of M1 macrophages, thus promoting enhancement of inflammation. However, the mechanisms by which TLRs regulate macrophage polarization in psoriasis are not well understood. In this study, we demonstrated that the TLR7/MyD88/NF‐κB signaling pathway plays a critical role in regulating the M1/M2 macrophage balance after IMQ treatment. Furthermore, previous studies have confirmed that activation of the TLR7(8)/MyD88/NF‐κB signaling pathway promotes NLRP3 inflammasome responses in keratinocytes following IMQ treatment [7, 9]. Here, we also found that activation of the TLR7(8)/MyD88/NF‐κB pathway is involved in regulating the proliferation and differentiation of keratinocytes in psoriatic lesions. GPR108 deficiency enhanced the activation of the TLR7/MyD88/NF‐κB signaling pathway in both macrophages and keratinocytes upon IMQ stimulation, compared to cells expressing wild‐type GPR108. This leads to the development of more severe psoriasiform lesions in IMQ‐induced mouse models.

**Figure 7:**
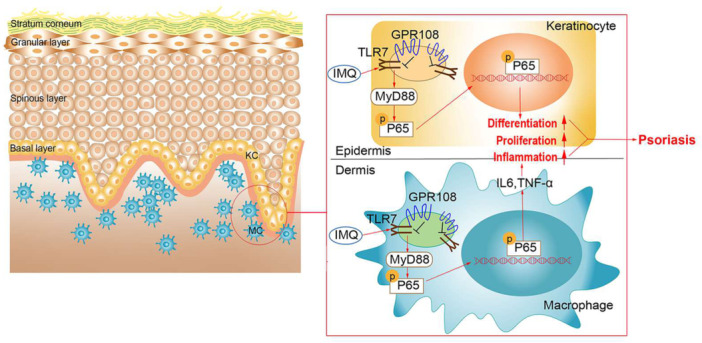
Schematic of GPR108's role in regulating TLR7 signaling in psoriasis. In an IMQ‐induced psoriasis‐like skin lesion model, the TLR7/MyD88/NF‐κB signaling pathway is activated in skin keratinocytes and macrophages. This activation leads to increased pro‐inflammatory cytokines, which in turn cause abnormal keratinocyte proliferation and differentiation, thereby exacerbating skin inflammation. GPR108 acts as a potential inhibitor of TLR7 signaling by competing with MyD88 for binding to TLR7. This competition inhibits the nuclear translocation of downstream NF‐κB, thus suppressing inflammation. Consequently, GPR108 deficiency enhances the activation of the TLR7/MyD88/NF‐κB signaling pathway and promotes psoriasis.

Given that GPR108 appears to mostly act as an inhibitor of inflammatory responses, the overexpression probably represents a homeostatic response to counter inflammation. GPR108 deficiency increases TLR‐induced proinflammatory cytokine production in mouse embryonic fibroblasts and macrophages [12]. GPR108 may play a vital role in maintaining a physiological balance between the stimulatory and inhibitory immune responses triggered by TLR7, as we have previously demonstrated that GPR108 exerts a negative regulation on TLRs‐triggered functions by antagonizing MyD88 [12, 32]. Therefore, the ineffective suppression of the excessive activation of the TLR7/NF‐κB signaling pathway by GPR108 may result in uncontrolled and progressively severe inflammatory responses of macrophages, as well as the proliferation and differentiation of keratinocytes during the progression of psoriasis (Figure 7).

In summary, our study identifies a novel role for GPR108 as a negative regulator of TLR7 signaling in psoriasis. This suggests that investigating pathways leading to GPR108 overexpression may yield new therapeutic principles for managing psoriatic diseases. Furthermore, developing small molecules that mimic GPR108's function, promote its expression, or stabilize its interaction with TLR7 could offer therapeutic avenues, with broader implications for other TLR‐driven inflammatory diseases.

## Author Contributions

**Wenwen Wang:** visualization, project administration, methodology, formal analysis, data curation, conceptualization, writing – original draft. **Yuyan Zhang:** resources, formal analysis, writing – original draft, visualization. **Kainan Liao:** methodology, formal analysis, writing – original draft, visualization. **Qiantong Xiang:** resources, formal analysis. **Dandan Zang:** validation, visualization. **Chunlin Cai:** formal analysis. **Fusheng Zhou:** formal analysis. **Haishen Zhou:** writing – original draft, writing – review and editing, conceptualization, resources, supervision, project administration, funding acquisition.

## Ethics Statement

All mouse experiments were approved by the Experimental Animal Ethics Committee of Anhui Medical University (approval no. LLSC 20200007).

## Conflicts of Interest

The authors declare no conflicts of interest.

## Supporting information


**Supporting Figure 1:** PCR analysis for the genotype of mice and immunoblotting identification of *GPR108* deletion in both HaCaT and THP‐1 Cell Lines. **Supporting Figure 2:** Flow cytometry analysis of T cells in peripheral blood of mice (*n* = 6/group). **Supporting Figure 3:** Heatmap diagram summarizing differential expression of the inflammation‐related genes in THP‐1 cells. **Supporting Table S1:** Oligonucleotides sequence.

## Data Availability

RNA‐seq Data are available at: https://www.ncbi.nlm.nih.gov/sra/PRJNA1028168.
