# Supplementary material for: GPR108 Negatively Regulates TLR7 Signaling in Imiquimod‐Induced Psoriasiform Dermatitis
Source: Immun Inflamm Dis. 2026 Feb 12;14(2):e70346. doi: 10.1002/iid3.70346 (PMC12902182; doi:10.1002/iid3.70346)
Supplement: Supplementary file 1 — Supporting Figure 1: PCR analysis for the genotype of mice and immunoblotting identification of GPR108 deletion in both HaCaT and THP‐1 Cell Lines. Supporting Figure 2: Flow cytometry analysis of T cells in peripheral blood of mice (n = 6/group). Supporting Figure 3: Heatmap diagram summarizing differential expression of the inflammation‐related genes in THP‐1 cells. Supporting Table S1: Oligonucleotides sequence. [file IID3-14-e70346-s001.docx]

**Title:**

GPR108 Negatively Regulates TLR7 Signaling in Imiquimod-Induced Psoriasiform Dermatitis.

**Authors:** Wang WW, *et al*.


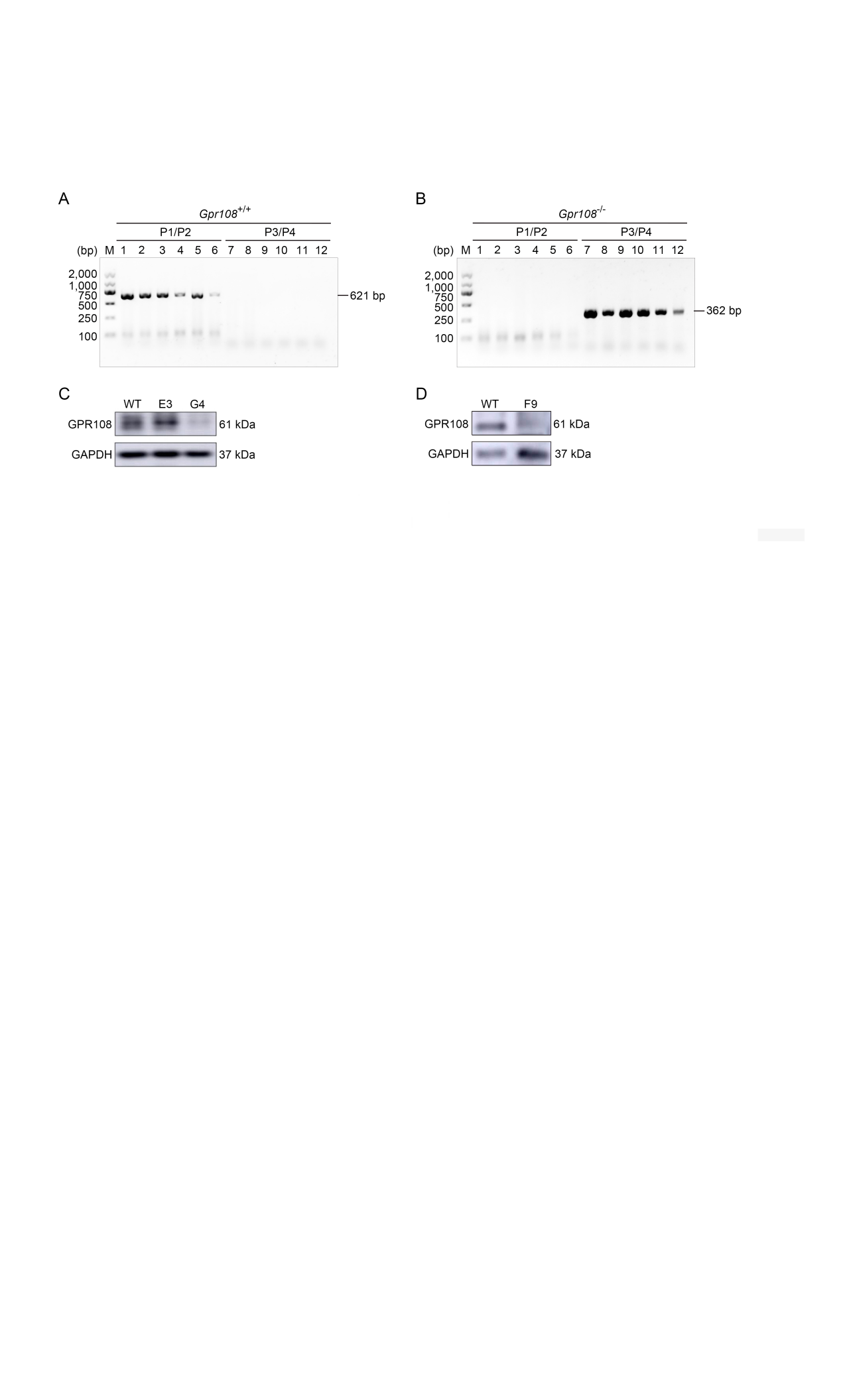


**Supplementary Fig. 1** PCR analysis for the genotype of mice and immunoblotting identification of *GPR108* deletion in both HaCaT and THP-1 Cell Lines. Deletion of the coding region of the *GPR108* gene in HaCaT and THP-1 cells using gRNAs (Table S1). The recombinant vectors with gRNAs and packaging plasmids, namely pMD2.G and psPAX2, were co-transfected into 293T cells to generate virus for infecting THP-1 and HaCaT cells. Single-cell clones of GFP-positive cells were obtained using a Flow Cytometer cell sorter (Beckman Coulter, MoFlo XDP) and then inoculated in 96-well plates with a cell density of one cell per well. The genomic DNA of these single-cell clones was collected, their PCR products were sequenced, and the expression of GPR108 in the cells was further assessed through Western blotting (WB). (A) PCR analysis fr the genomic DNA extracted from the tails of mice. The primers specific to the wt mouse *Gpr108* (P1/P2) are listed in supplementary Table S1. (B) PCR analysis for the genome DNA derived from the tails of mice. The primers of the mouse *Gpr108* deletion (P3/P4) are listed in the supplementary Table S1. (C) WB analysis of GPR108 expression in HaCaT cells. WT indicates HaCaT cells; G4 and E3 denote the number of the signal *GPR108*^-/-^ HaCaT clones. (D) WB analysis of GPR108 expression in THP-1 cells. WT denotes the THP-1 cells. F9 indicates the F9 single cell clone with *GPR108* deletion.

M, DNA marker; No. 1-12, represents the numbers of mice.

**
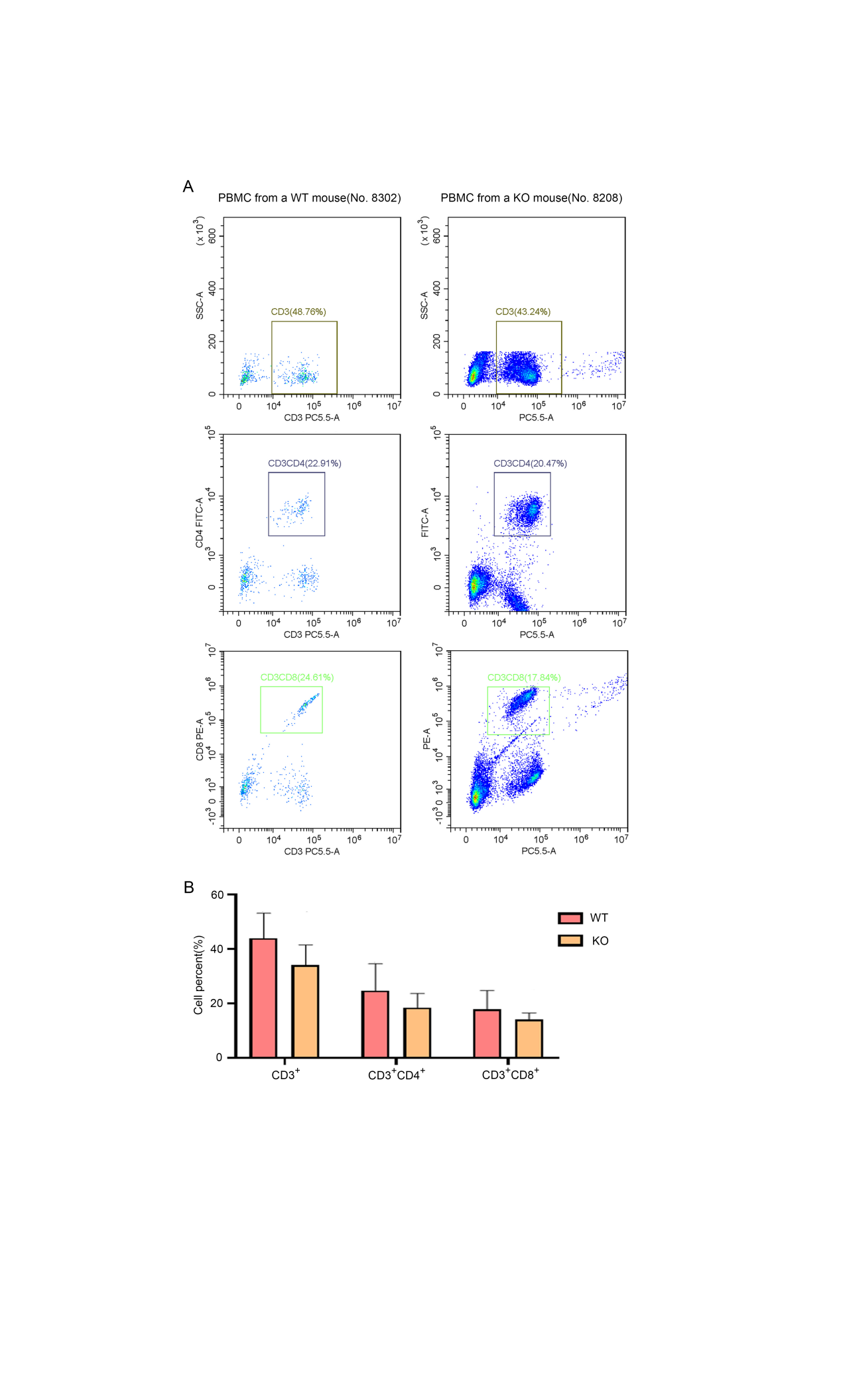
**

**Supplementary Fig. 2** Flow cytometry analysis of T cells in peripheral blood of mice (n= 6/group). Peripheral blood monocytes (PBMC) were isolated from mice and stained with CD3-Per CP-Cy5.5, and CD4-FITC, CD8-PE. No differences were observed in the percentages of CD3+, CD3+CD4+, and CD3+CD8+ T cells between the wild-type (WT) and knockout (KO) mice.


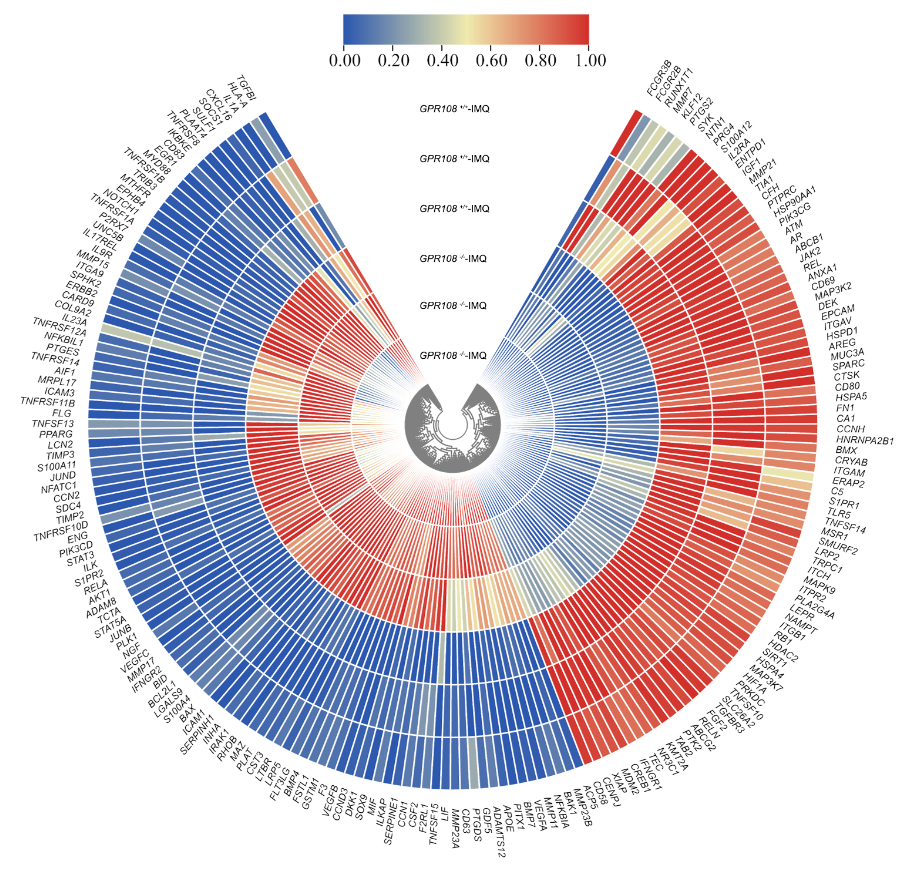


**Supplementary Fig. 3** Heatmap diagram summarizing differential expression of the inflammation-related genes in THP-1 cells. RNA-seq analysis was conducted on both *GPR108*^-/-^ and *GPR108*^+/+^ THP-1 cells following a 48-hour exposure to IMQ at a concentration of 10 µg/ml. There is a total of 195 inflammation-related genes that exhibited differential expression in GPR108-deficient cells compared to WT cells. Among these, 55 genes displayed upregulated expression, while 23 genes showed downregulated expression.

**Supplementary Table. 1**

Table S1. Oligonucleotides sequence.

| Oligos | Sequence（5’ to 3’） | Aims |
| --- | --- | --- |
| Human sgRNA1 (FW) | CACCGCTCACCAGACTACGGGGAAC | gRNAs for *GPR108* deletion by using the CRISPR/Cas9 in HaCaT and THP-1 cells. |
| Human sgRNA1 (RW) | AAACGTTCCCCGTAGTCTGGTGAGC |  |
| Human sgRNA2 (FW) | CACCGGGACAGCACCGGTTCCGAA |  |
| Human sgRNA2 (RW) | AAACTTCGGAACCGGTGCTGTCCC |  |
| Human *GPR108* (FW) | AGCCTGGGGTCATAGGTCAT | PCR identification  of GPR108 deletion in human cells. |
| Human *GPR108* (RW) | AGGGCAGAGAGGCCAGATAA |  |
| Mouse *Gpr108* wt (P1) | TGAGATTGGGAGGAGTTTGG | PCR identification of *Gpr108* deletion in mice |
| Mouse *Gpr108* wt (P2) | CTGCCTCGGACTGAAGTAGG |  |
| Mouse *Gpr108* mut (P3) | ATTGTACCACAAGAGGCTGAACTG |  |
| Mouse *Gpr108* mut (P4) | ACTATTGGCTGAAAAGTGGGTAGG |  |
| Human *IL-10* (FW) | TTCCATTCCAAGCCTGACCAC | qPCR detection |
| Human *IL-10* (RW) | GCTCCCTGGTTTCTCTTCCTAAG |  |
| Human *GAPDH* (FW) | GTATCGTGGAAGGACTCATGAC | qPCR detection |
| Human *GAPDH* (RW) | ACCACCTTCTTGATGTCATCAT |  |
| Human *TNF-α* (FW) | CACTTCGAAACCTGGGATTCAG | qPCR detection |
| Human *TNF-α* (RW) | GGTCTCCAGATTCCAGATGTCAG |  |
| Human *TGF-β* (FW) | TACCTGAACCCGTGTTGCTCTC | qPCR detection |
| Human *TGF-β* (RW) | GTTGCTGAGGTATCGCCAGGAA |  |
| Human *IL6* (FW) | AGACAGCCACTCACCTCTTCAG | qPCR detection |
| Human *IL6* (RW) | TTCTGCCAGTGCCTCTTTGCTG |  |
